# Rutin and Quercetin Decrease Cholesterol in HepG2 Cells but Not Plasma Cholesterol in Hamsters by Oral Administration

**DOI:** 10.3390/molecules26123766

**Published:** 2021-06-21

**Authors:** Ning Liang, Yuk-Man Li, Zouyan He, Wangjun Hao, Yimin Zhao, Jianhui Liu, Hanyue Zhu, Erika Kwek, Ka-Ying Ma, Wen-Sen He, Zhen-Yu Chen

**Affiliations:** 1 School of Life Sciences, Chinese University of Hong Kong, Shatin, NT, Hong Kong 999077, China; liangning1234@126.com (N.L.); charis_li@hotmail.com (Y.-M.L.); hezouyan1017@gmail.com (Z.H.); hwjcuhk@hotmail.com (W.H.); yimin_zhao@outlook.com (Y.Z.); joyce91ha@gmail.com (J.L.); zhuhanyue29@gmail.com (H.Z.); erika.kwek@hotmail.com (E.K.); rubyma06@cuhk.edu.hk (K.-Y.M.); 2 College of Physics and Optoelectronic Engineering, Shenzhen University, Shenzhen 518060, China; 3 Health Science Center, Shenzhen Key Laboratory of Novel Natural Health Care Products, Engineering Laboratory of Shenzhen Natural Small Molecule Innovative Drugs Innovation Platform for Natural Small Molecule Drugs, Department of Pharmacy, School of Medicine, Shenzhen University, Shenzhen 518060, China; 4 School of Food and Biological Engineering, Jiangsu University, 301 Xuefu Road, Zhenjiang 212013, China; wshe2013@163.com

**Keywords:** rutin, quercetin, cholesterol, hamsters, LXR, SREBP2, LDLR

## Abstract

Rutin (R) and quercetin (Q) are two widespread dietary flavonoids. Previous studies regarding the plasma cholesterol-lowering activity of R and Q generated inconsistent results. The present study was therefore carried out to investigate the effects of R and Q on cholesterol metabolism in both HepG2 cells and hypercholesterolemia hamsters. Results from HepG2 cell experiments demonstrate that both R and Q decreased cholesterol at doses of 5 and 10 µM. R and Q up-regulated both the mRNA and protein expression of sterol regulatory element binding protein 2 (SREBP2), low-density lipoprotein receptor (LDLR), and liver X receptor alpha (LXRα). The immunofluorescence study revealed that R and Q increased the LDLR expression, while only Q improved LDL-C uptake in HepG2 cells. Results from hypercholesterolemia hamsters fed diets containing R (5.5 g/kg diet) and Q (2.5 g/kg diet) for 8 weeks demonstrate that both R and Q had no effect on plasma total cholesterol. In the liver, only Q reduced cholesterol significantly. The discrepancy between the in vitro and in vivo studies was probably due to a poor bioavailability of flavonoids in the intestine. It was therefore concluded that R and Q were effective in reducing cholesterol in HepG2 cells in vitro, whereas in vivo, the oral administration of the two flavonoids had little effect on plasma cholesterol in hamsters.

## 1. Introduction

Hypercholesterolemia is a major cause of cardiovascular diseases. Healthy diets and an active lifestyle can improve the plasma lipid profile by reducing blood total cholesterol (TC) and low-density lipoprotein cholesterol (LDL-C) while elevating high-density lipoprotein cholesterol (HDL-C) [1,2]. Cholesterol homeostasis is regulated by its absorption, synthesis, conversion, and excretion. In the lumen of the small intestine, cholesterol is transported into enterocytes, where it is esterified and packed into chylomicrons followed by entering the lymphatic system [3,4]. Synthesis of cholesterol is mainly carried out in the liver, starting with acetyl CoA, mediated by regulation of the activity of 3-hydroxy-3-methylglutaryl-CoA reductase (HMG-CoA R). The LDL receptor (LDLR) is responsible for the removal of cholesterol from the circulation and lowering plasma LDL-C [5,6]. Sterol regulatory element binding protein 2 (SREBP2) governs the expression of both HMG-CoA R and LDLR. Cholesterol 7α-hydroxylase (CYP7A1) is a key enzyme for converting excessive cholesterol to bile acids in the liver [7]. Another regulator is liver X receptor α (LXRα), which regulates the encoding of CYP7A1 [8].

**Figure 1:**
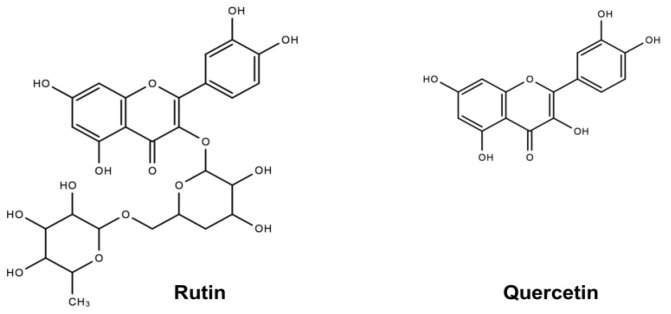
Chemical structures of rutin and quercetin.

Rutin (R) is a natural flavonoid present in many foods [9,10,11] (Figure 1). R has been reported to possess several biological functions, including anti-oxidative, anti-diabetic, anti-adipogenic, anti-inflammatory, and anti-cancer activities [9,12,13,14]. R is structurally composed of quercetin (Q) as its aglycone and rutinose as its sugar moiety [15] (Figure 1). As with R, Q is also widely present in many foods, such as buckwheat, apple, onion, red wine, and cranberry [16,17,18]. Q has also been demonstrated to possess anti-oxidative, anti-viral, anti-atherosclerotic, and anti-inflammatory activities [19,20,21,22,23]. Various studies have been carried out to investigate the effects of R and Q on cholesterol metabolism using different models, including vascular endothelial cells, intestinal epithelial cells, macrophages, hepatic cells, male Swiss ablino mice, male Wistar rats, male Sprague Dawley rats, male New Zealand white rabbits, male C576J/BL mice, and male golden Syrian hamsters [24,25,26,27]. However, these studies generated inconsistent results regarding the cholesterol-lowering activity of R and Q. We had previously shown that consumption of rutin-rich buckwheat was effective in reducing plasma cholesterol in hypercholesterolemia hamsters; however, R and Q appeared not to be the active ingredients [16]. This arouses our interest to further investigate if R and Q act differently in vitro and in vivo in affecting cholesterol metabolism.

The objectives of the present study were to (i) study the effect of R and Q on cholesterol metabolism using HepG2 cells as an in vitro model; (ii) examine the effect of R and Q on blood cholesterol using hamsters as an in vivo model; and (iii) investigate the interaction of R and Q with the expression of genes involved in cholesterol metabolism. We chose hamsters as an in vivo model because hamsters are relatively hyper-responsive to dietary cholesterol, excrete bile acids, and synthesize cholesterol and bile acids in a manner similar to that in humans [28,29].

## 2. Results

### 2.1. Effects on Cell Viability

**Figure 2:**
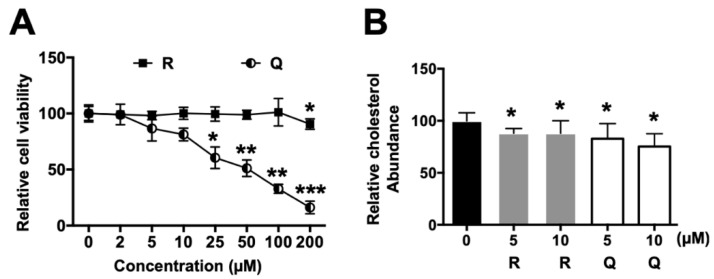
(**A**) Cytotoxicity of rutin (R) and quercetin (Q). HepG2 cells were incubated with R or Q for 24 h; thereafter, cell viability was measured by MTT assay. (**B**) Changes in total cholesterol content in HepG2 cells with incubation of R or Q. Each value represents the mean ± standard deviation from 3 representative experiments. *** *p* < 0.005; ** *p* < 0.01; or * *p* < 0.05 compared with vehicle group.

To estimate the cytotoxicity of R and Q, HepG2 cells were incubated with various doses of R and Q for 24 h. The results indicate that R and Q exhibited a different cytotoxicity in HepG2 cells. R did not affect cell viability at concentrations ranging from 3 to 100 µM, while Q had a significant toxicity in HepG2 cells from 25 µM onwards (Figure 2A). Therefore, the effects of R and Q on HepG2 cells were evaluated at 5 and 10 µM.

### 2.2. Cholesterol Content in HepG2 Cells

The cholesterol content in HepG2 cells was quantified using a cholesterol liquicolor test. R and Q markedly reduced the cholesterol content in HepG2 cells at doses of 5 and 10 µM compared with the vehicle group (* *p* < 0.05, Figure 2B). The results from the in vitro experiment reveal that R and Q could modulate cholesterol metabolism.

### 2.3. mRNA and Protein Abundances in HepG2 Cells

**Figure 3:**
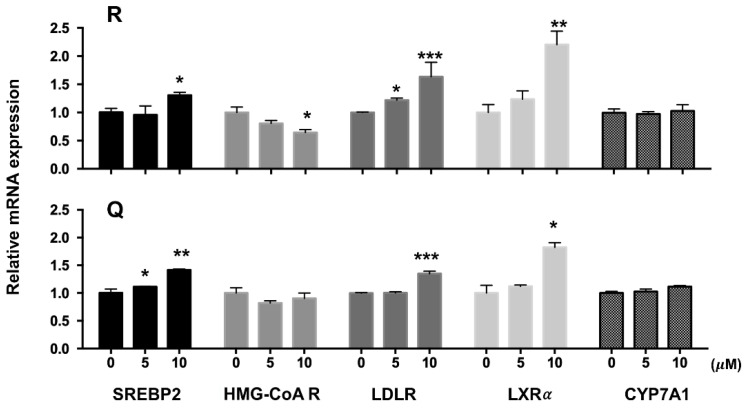
Effects of rutin (R) and quercetin (Q) on mRNA of SREBP2, HMG-CoA R, LDLR, and LXRα in HepG2 cells as determined by real-time PCR. HepG2 cells were incubated with 5 and 10 µM of R and Q for 24 h. Data were normalized with GAPDH. Values were expressed as means ± standard deviations (n = 3). *** *p* < 0.005; ** *p* < 0.01; or * *p* < 0.05 compared with the vehicle group.

**Figure 4:**
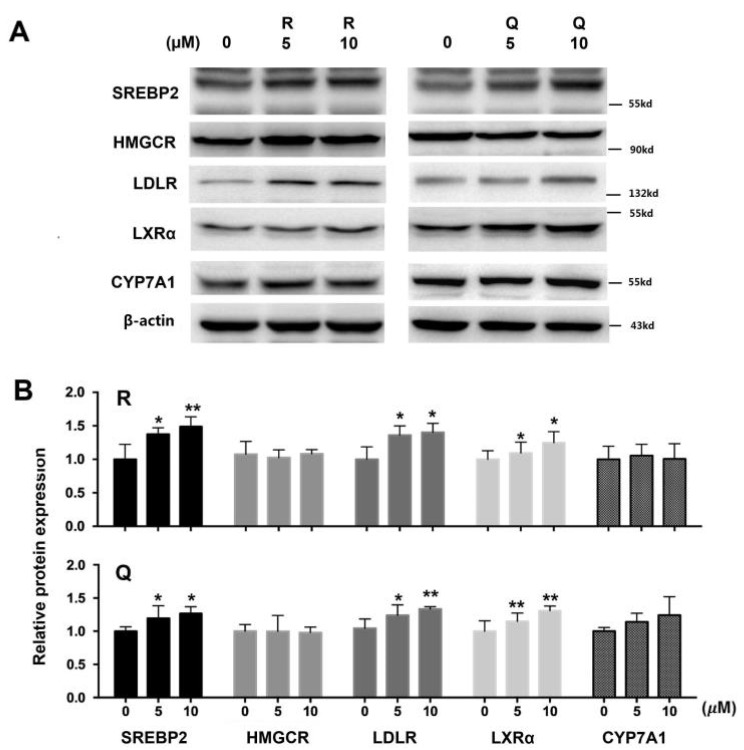
Effects of rutin (R) and quercetin (Q) on protein mass of SREBP2, HMG-CoA R, LDLR, LXRα, and CYP7A1 in HepG2 cells treated with 5 and 10 µM of R and Q for 24 h. (**A**) The total protein was extracted and subjected to Western blot analyses. (**B**) The relative band intensity was quantified and normalized with β-actin. Data represent the means ± standard deviations (n = 3). ** *p* < 0.01 or * *p* < 0.05 compared with the vehicle group.

mRNA levels of SREBP2, LDLR, and LXRα in HepG2 cells treated with 10 µM of R and Q were up-regulated significantly, whereas R at a dose of 10 µM down-regulated the mRNA of HMG-CoA R (Figure 3). R and Q had no significant effects on mRNA levels of CYP7A1 (Figure 3). R and Q treatments increased the protein abundances of SREBP2 (the nuclear active fragment), LDLR, and LXRα. However, they had no significant effects on the protein mass of HMG-CoA R and CYP7A1 (Figure 4).

### 2.4. Fluorescence Immunostaining of LDLR and LDL-C Uptake

**Figure 5:**
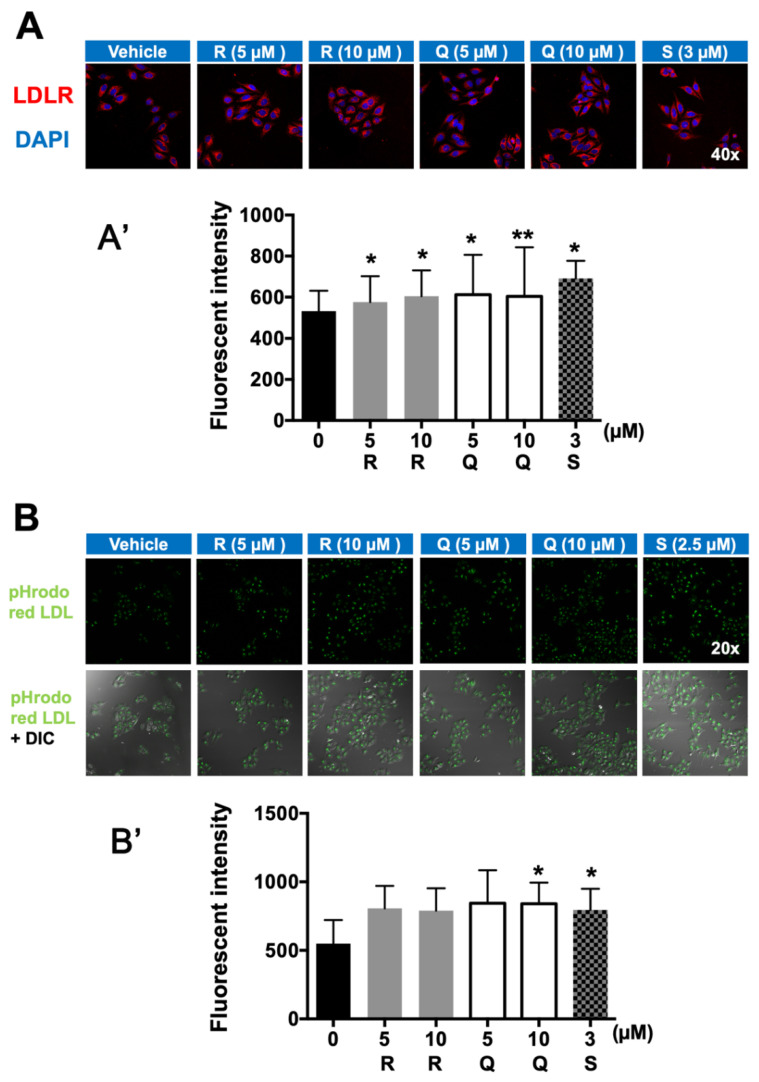
Effects of rutin (R) and quercetin (Q) on LDLR expression (**A**,**A’**) and LDL-C uptake (**B**,**B’**) determined by using an immunofluorescence staining method. Starved HepG2 cells were incubated with R, Q, or S (simvastatin) for 24 h and then immunostained with an anti-LDLR antibody. (**A**) Images of LDLR (red) and the merged images with DAPI (blue) are shown with magnification at ×400; the fluorescence intensity was quantified (**A’**). (**B**) The HepG2 cells were starved in medium containing R and Q for 8 h and then stimulated with pHrodo red LDL-C (green) for 3 h. The photos were taken by confocal microscope with magnification at ×200; the fluorescence intensity was quantified (**B’**). Values are expressed as means of the cellular fluorescence intensity ± standard deviations (n = 50) compared with those for the control group. ** *p* < 0.01 or * *p* < 0.05 compared with the vehicle group.

Consistent with the up-regulation of LDLR expression in HepG2 cells at both the mRNA and protein levels (Figure 3 and Figure 4), the fluorescence immunostaining clearly visualized that both R and Q up-regulated the amount of LDLR in HepG2 cells (Figure 5A). HepG2 cells were covered with exogenous pHrodo red LDL-C to determine the effect of R and Q on LDL-C endocytosis. The data demonstrated that R and Q could enhance the uptake of pHrodo red LDL-C; however, only the effect of Q was statistically significant (Figure 5B).

### 2.5. Plasma Profile and Liver Cholesterol

**Figure 6:**
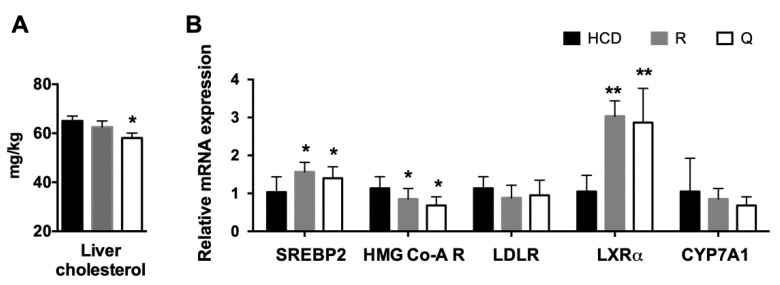
Effects of rutin (R) and quercetin (Q) on cholesterol modulation in hamsters fed a high-cholesterol control diet (HCD) or one of two experimental diets containing R and Q. At week 8: (**A**) liver cholesterol; and (**B**) mRNA expression level of cholesterol-related genes of the hamsters. Data were normalized with GAPDH. Values are expressed as means ± standard deviations (n = 8). ** *p* < 0.01; or * *p* < 0.05 compared with the control group.

Golden Syrian hamsters were selected to be an in vivo model for investigating the effect of R and Q on blood cholesterol. Part of the study on blood cholesterol had been reported in our previous publication [16]. Supplementation of R (5.5 g/kg diet) and Q (2.5 g/kg diet) did not affect the body weight gain compared with the control diet. Additionally, there was no statistical difference in the plasma TC level in the R or Q group compared with the control group as we had previously reported [16]. Similar to that in the HepG2 cell experiment, feeding with the Q diet significantly reduced hepatic cholesterol. However, feeding with the R diet had no effect on liver cholesterol (Figure 6A).

### 2.6. mRNA Abundance of Target Genes in the Liver

The hepatic mRNA expression level of the related genes was quantified. Feeding with R and Q diets markedly up-regulated the mRNA levels of LXRα and SREBP2, whereas they down-regulated the HMG-CoA R mRNA level. Feeding with R and Q diets had no effect on the mRNA of LDLR and CYP7A1 (Figure 6B).

## 3. Discussion

An abnormal elevation in blood cholesterol is a key risk factor for cardiovascular diseases [30]. Dietary modulation of blood cholesterol is an efficient way to reduce the risk of atherosclerosis and cardiovascular diseases [17]. As a part of our study investigating the effect of rutin-rich buckwheat on plasma cholesterol and identifying its active ingredient, the present study examined the effects of R and Q on cholesterol metabolism in both HepG2 cells and golden Syrian hypercholesterolemia hamsters. 

In HepG2 cells, R and Q could significantly reduce the cholesterol concentration (Figure 2). The effect of R and Q on the gene expression of SREBP2, LDLR, and HMG-CoA R in in vitro HepG2 cells was not always consistent with that observed in the in vivo hamster model. Research has shown that SREBP2 regulates the gene expression of not only LDLR but also HMG-CoA R [31,32]. Generally, LDLR removes plasma cholesterol into the liver or other tissue via mediating cholesterol uptake, while HMG-CoA R is a key enzyme in cholesterol synthesis. It is known that intracellular cholesterol regulates the transcription of LDLR via a negative feedback mechanism. At a lower cellular cholesterol environment, SREBP2 maturation will be enhanced and activate the expression of LDLR [6]. In the present study, mRNA abundance analysis in the liver and HepG2 cells showed that R and Q could up-regulate the expression of SREBP2 (Figure 3 and Figure 6). It was also found that LDLR mRNA and protein abundances were both enhanced by R and Q in HepG2 cells (Figure 3 and Figure 4). This was in agreement with the report of Moon et al., who found that Q increased the expression of SREBP2 and LDLR [33]. This was also confirmed in the present study where Q was capable of enhancing LDL-C endocytosis (Figure 5B). However, R and Q supplementation into diets of hamsters had no effect on the mRNA of LDLR. Feeding with R and Q diets down-regulated the mRNA of hepatic HMG-CoA R (Figure 6); however, in HepG2 cells, incubation of R and Q had no effect on the mRNA of HMG-CoA R (Figure 3 and Figure 4). The present study did not study the effect of R and Q on the activity of HMG-CoA R in the liver of hamsters. However, computer docking has shown that R had a strong affinity with HMG-CoA R, and Q could significantly reduce the activity of HMG-CoA R [34,35].

LXRα is highly expressed in metabolically active tissues and organs, including adipose tissue, macrophages, the intestine, and the liver [36]. LXRα signaling regulates lipid homeostasis by activating the downstream molecules. It is reported that LXRα is capable of regulating CYP7A1 expression at the transcriptional level [8]. The present study found that R and Q could up-regulate the mRNA level of LXRα in both HepG2 cells and the liver of hamsters; however, no effect of R and Q on the mRNA of CYP7A1 was seen. The present results show that R and Q significantly decreased the cholesterol in HepG2 cells, while they had a down-trend effect on liver cholesterol in the hamster liver even though it was not statistically significant. As LXRα is a sensor of cholesterol in the liver, it is possible that cholesterol reduction in HepG2 cells or liver cells led to up-regulation of LXRα. CYP7A1 is an enzyme regulating the conversion of cholesterol to bile acids in the liver if hepatic cholesterol is excessive. In the present study, mRNA CYP7A1 remained unchanged, while R and Q decreased the cholesterol in HepG2 and the liver of hamsters. 

In the in vivo golden Syrian hamster model, Q decreased liver cholesterol, whereas R had no effect on liver cholesterol. Both R and Q had no effect on plasma TC. As we had reported, plasma TC in R- and Q-supplemented hamsters was 241 ± 23 and 250 ± 25 mg/dL, respectively, values which were not statistically different from the control value (259 ± 41 mg/dL), indicating R and Q had little effect on blood cholesterol in vivo [16]. 

The in vivo study was in contrast to the report of Mariee et al., who found that administration of Q at 15 mg/kg body weight in Sprague Dawley rats for two weeks reduced plasma TC by 20% [37]. In another study conducted in male Wistar rats, R at a dose of 0.2% could reduce plasma TC by 22% [11]. The differences in results among the various in vitro and in vivo studies are most likely attributable to the different animal models used and the poor bioavailability of both R and Q [38,39,40].

The physical and metabolic properties of R and Q result in poor solubility, which may affect their biochemical efficacy [41,42]. As reported, quercetin can be absorbed as its native form in the stomach and small intestine [43]. Once rutin is ingested, it will undergo hydrolysis by glycosidase in the colon, releasing Q and rutinoside, meaning the absorption of R takes more time. Several flavonoid metabolites were detected in human plasma, including tamarixetin, isorhamnetin, and kaempferol; however, their quantity is much lower than quercetin in plasma. In plasma, the major circulating metabolites of Q are conjugated with glucuronide, methyl, or sulfates, such as quercetin 3-*O*-β-D-glucuronide (Q3GA) and quercetin-3’-sulfate [44]. Thus, physiologically speaking, these phase-II metabolites, instead of R/Q, enter into the circulation system and exert their biological functions [45]. Therefore, mimicking the physiological in vivo conditions, in order to study the function of these circulating metabolites at the cell culture level, will contribute to unveiling the mode of action of R and Q at the in vivo level.

Based on our data and the characters of R and Q, we speculated that the weak bioactivity in hamsters was probably attributable to their poor bioavailability and solubility. Much effort has been devoted to improving their solubility and absorption [46,47,48]. By utilizing nano-lipid complexes, the solubility of rutin was significantly enhanced over 20 times, accompanied by a relatively higher absorption and oral bioavailability, in rats [48]. Similarly, in a clinical trial, quercetin in a food-grade lecithin-based formulation was found to provide a significant improvement in bioavailability [46]. Enhancing the bioavailability of rutin and quercetin may bring the two natural flavonoids to the forefront of dietary supplementation in the management of hypercholesterolemia.

In summary, R and Q could favorably modulate cholesterol metabolism in HepG2 cells, through up-regulation of SREBP2, LDLR, and LXRα. However, dietary supplementation of R and Q had little significant effect on plasma total cholesterol in hypercholesterolemia hamsters. The present study does not fully support the claim that oral supplementation of R and Q in diets is hypocholesterolemic in vivo. In consideration of the poor solubility and bioavailability, application of better delivery systems or investigation of related metabolites may be conducive to facilitating the effective utilization of R and Q as dietary supplements in the treatment of hypercholesterolemia.

## 4. Materials and Methods

### 4.1. Chemicals and Reagents

Q and simvastatin were obtained from Sigma (St. Louis, MO, USA). R was purchased from DND Pharm-technology Co. Inc. (Shanghai, China). The antibodies against β-actin (sc-69879, 1:3000 dilution) and SREBP2 (sc-13552, 1:750 dilution), and anti-mouse and anti-rabbit horseradish perioxidase (HRP)-conjugated secondary antibodies were purchased from Santa Cruz Biotechonology (Dallas, TX, USA). The antibodies against LXRα (ab176323, 1:1000 dilution), LDLR (ab52818, 1:500 dilution), and CYP7A1 (ab65596, 1:1000 dilution) were purchased from Abcam (Cambridge, UK). The anti-HMG-CoA R (ABS229, 1:2000 dilution) antibody was bought from Millipore (Burlington, MA, USA). The immunofluorescence secondary antibody and Alexa Fluor 488 Goat anti-Rabbit IgG (H + L) were purchased from Thermo (Waltham, MA, USA).

### 4.2. Cell Culture

HepG2 cells were purchased from the American Type Culture Collection (ATCC, Manassas, VA, USA) [49]. HepG2 cells were cultured in RPMI-1640 (Gibco, Rockville, MD, USA), supplemented with 10% fetal bovine serum (FBS) and 1% antibiotic-antimycotic. The cells were cultured at 37 °C and 5% CO_2_ humidified atmosphere.

### 4.3. MTT Assay

The cytotoxicity of R and Q on cells was evaluated by MTT assay as described previously [50]. Briefly, cells were cultured in 96-well plates overnight. Then, cells were treated without or with addition of R and Q, ranging 3 to 200 μM, for 24 h, followed by staining with 30 μL MTT (5 mg/mL) for another 4 h. The medium was then replaced with 200 μL DMSO. After 10 min of complete dissolution, the cell viability was quantified by measuring the optical density at 570 nm using a UV–Vis spectrophotometer (Genesys 5, Spectronic Instruments, New York, NY, USA). 

### 4.4. Cholesterol Determination in Cells

HepG2 cells (5 × 10^5^ cells/well) were equally seeded in 6-well plates. The adherent cells were exposed to R and Q for 24 h. Then, the cells were extracted using a mixture of chloroform and methanol (3:1, *v*/*v*) for 30 min, the samples were then centrifuged at 13,000× *g* × 15 min for purification. The pellet was lysed in a RIPA buffer for measurement of protein abundance, and the supernatant was collected for total cholesterol measurement by a cholesterol liquicolor test (Stanbio, Boerne, TX, USA) using a microplate reader. 

### 4.5. Real-Time PCR Analysis

Quantitative real-time PCR assay was performed as previously described [51]. Briefly, total RNA was isolated from HepG2 cells or hamster tissue by using Trizol reagent (Invitrogen, Carlsbad, CA, USA). Complementary DNA was synthesized from RNA with a high-capacity cDNA reverse transcription kit. Real-time PCR analysis was conducted on a StepOnePlus Real-time PCR System (Applied Biosystems, Foster City, CA, USA) using SYBR Green Fast Universal PCR Master Mix; the primer sequences are shown in Table 1. The mRNA level of the markers was normalized with that of GAPDH.

### 4.6. Western Blotting

HepG2 cells (5 × 10^5^ cells/dish) were distributed in 100 mm culture dishes for 24 h. After 6 h of starvation, cells were incubated with cell growth medium containing R and Q for 24 h. Cell lysates were collected, and total protein was electrophoretically separated and transferred onto a PVDF membrane as described previously [49]. The membrane was incubated with primary antibody and HRP-conjugated relative secondary antibodies. Protein bands were visualized with Pierce ECL substrate and detected with the ChemiDoc Touch Gel Imaging System (Bio-Rad, Hercules, CA, USA); subsequently, the relative band intensity was quantified. 

### 4.7. LDL Uptake Assay

1 × 10^5^ HepG2 cells were cultured in 35 mm glass-bottom cell culture dish plates overnight [46]. Then, cells were starved in a serum-free medium containing 0.5% BSA for 6 h and incubated with R and Q for 8 h; thereafter, the medium was removed, and cells were covered with 10 ug/mL pHrodo red LDL for 3 h. Subsequently, the cells were washed twice, and the images were captured on a confocal microscope (Olympus FV1000 ix81, Shinjuku, Tokyo, Japan).

### 4.8. Immunofluorescence Staining

HepG2 cells were cultured in glass-bottom cell culture plates at 37 °C for 12 h. After 6 h of starvation with a serum-free medium, cells were overlaid with medium containing R and Q for another 24 h. Following this, the cells were washed with PBS, fixed with paraformaldehyde (4%) for 15 min at room temperature, and permeabilized with Triton X-100 (0.1%) for 5 min, and then cells were blocked in BSA (5% in PBS) for at least 1 h and incubated with antibody against LDLR (1:250 in BSA) overnight at 4 °C. After being washed with PBS three times, cells were immunostained with secondary fluorescence antibody for 1 h at room temperature [52]. The cell nucleus was visualized with DAPI. After washing with PBS three times, cells were examined using a confocal microscopy, and the fluorescence intensity was analyzed with Fluoview viewer software (Olympus) [47].

### 4.9. Hamsters and Diets

Three diets were prepared as a part of our buckwheat study reported previously [16]. A high-cholesterol control diet (HCD) was prepared by mixing the following ingredients of 508 g corn starch, 242 g casein, 119 g sucrose, 50 g lard, 40 g mineral mix, 20 g vitamin mix, 1 g DL-methionine, and 2 g cholesterol. Diets were prepared by adding 8.2 mmol (5.5 g·kg^−1^, human equivalent dose = 0.74 g·kg^−1^) of R and 8.2 mmol (2.5 g·kg^−1^, human equivalent dose = 0.34 g·kg^−1^) of Q into one kilogram of the HCD diet. Male golden Syrian hamsters were divided into three groups (n = 8) and housed in wire-bottom cages at 23 °C with a 12 h light–dark cycle in an animal room. They were fed one of three diets, namely, HCD control diet, R diet, or Q diet, for 8 weeks. Blood sample was collected from the retro-orbital sinus at the end of week 8 after overnight fasting. After the 3-day recovery, all hamsters were sacrificed under carbon dioxide suffocation. Plasma TC and HDL-C were measured using the commercial enzymatic kits from Infinity (Waltham, MA, USA) and Stanbio Laboratories (Boerne, TX, USA), respectively. The experimental protocols were approved by the Animal Experimental Ethical Committee, The Chinese University of Hong Kong (Ref. No. 15-066-MIS).

### 4.10. Liver Cholesterol Determination

Cholesterol in the liver was determined according to a previously described method [53]. In brief, all liver lipids were extracted using a solvent mixture of chloroform–methanol (2:1, *v*/*v*) with the addition of 5α-cholestane as an internal standard. After saponification, the remaining cholesterol was extracted into cyclohexane, followed by converting to its TMS-ether derivative. The analysis of cholesterol TMS-ether derivatives was carried out on a SAC-5 capillary column (30 m × 0.25 mm, Supelco, Bellefonte, PA, USA) in a Shimadzu GF-14B equipped with an FID detector. The amount of cholesterol in the liver was calculated according to the internal standard added.

### 4.11. Statistical Analyses

All values were analyzed using one-way analysis of variance (ANOVA) followed by a post hoc LSD analysis to detect any significant differences between any two groups. Data were expressed as mean ± standard deviation (SD). Significance was defined as a *p*-value less than 0.05.

## Figures and Tables

**Table 1:** Real-time PCR primer sequences.

Gene	For HepG2 Cells	For Hamster
GAPDH	Fw: CCCACTCCTCCACCTTTGAC	Fw: GAACATCATCCCTGCATCCA
	Rv: TCTTCCTCTTGTGCTCTTGC	Rv: CCAGTGAGCTTCCCGTTCA
SREBP2	Fw: AACGGTCATTCACCCAGGTC	Fw: GGACTTGGTCATGGGAACAGATG
	Rv: GGCTGAAGAATAGGAGTTGCC	Rv: TGTAATCAATGGCCTTCCTCAGAAC
HMG-CoA R	Fw: TGATTGACCTTTCCAGAGCAAG	Fw: CGAAGGGTTTGCAGTGATAAAGGA
	Rv: CTAAAATTGCCATTCCACGAGC	Rv: GCCATAGTCACATGAAGCTTCTGTA
LDLR	Fw: ACGGCGTCTCTTCCTATGACA	Fw: GCCGGGACTGGTCAG ATG
	Rv: CCCTTGGTATCCGCAACAGA	Rv: ACAGCCACCATTGTTGTCCA
LXRα	Fw: TCTGGAGACATCTCGGAGGTA	Fw: GTTTGTCCTGAGCTTCGTCC
	Rv: GGCCCTGGAGAACTCGAAG	Rv: CACCGCTGTGGCAAACATAG
CYP7A1	Fw: GCAATTTGGTGCCAATCCTCT	Fw: GGTAGTGTGCTGTTGTATATGGGTTA
	Rv: GCACAACACCTTATGGTATGACA	Rv: ACAGCCCAGGTATGGAATCAAC

## Data Availability

Data are contained within the article.

## Sample Availability

Samples are not available from the authors.
